# Calcium Oxalate Crystals: An Integral Component of the *Sclerotinia sclerotiorum/Brassica carinata* Pathosystem

**DOI:** 10.1371/journal.pone.0122362

**Published:** 2015-03-27

**Authors:** Margaret B. Uloth, Peta L. Clode, Ming Pei You, Martin J. Barbetti

**Affiliations:** 1 School of Plant Biology, Faculty of Science, The University of Western Australia, 35 Stirling Highway, Crawley, WA, 6009, Australia; 2 Centre for Microscopy, Characterisation and Analysis, Faculty of Science, The University of Western Australia, 35 Stirling Highway, Crawley, WA, 6009, Australia; 3 School of Plant Biology and The UWA Institute of Agriculture, Faculty of Science, The University of Western Australia, 35 Stirling Highway, Crawley, WA, 6009, Australia

## Abstract

Oxalic acid is an important virulence factor for disease caused by the fungal necrotrophic pathogen *Sclerotinia sclerotiorum*, yet calcium oxalate (CaOx) crystals have not been widely reported. *B*. *carinata* stems were infected with *S*. *sclerotiorum* and observed using light microscopy. Six hours post inoculation (hpi), CaOx crystals were evident on 46% of stem sections and by 72 hpi on 100%, demonstrating that the secretion of oxalic acid by *S*. *sclerotiorum* commences before hyphal penetration. This is the first time CaOx crystals have been reported on *B*. *carinata* infected with *S*. *sclerotiorum*. The shape of crystals varied as infection progressed. Long tetragonal rods were dominant 12 hpi (68% of crystal-containing samples), but by 72 hpi, 50% of stems displayed bipyramidal crystals, and only 23% had long rods. Scanning electron microscopy from 24 hpi revealed CaOx crystals in all samples, ranging from tiny irregular crystals (< 0.5 μm) to large (up to 40 μm) highly organized arrangements. Crystal morphology encompassed various forms, including tetragonal prisms, oval plates, crystal sand, and druses. Large conglomerates of CaOx crystals were observed in the hyphal mass 72 hpi and these are proposed as a strategy of the fungus to hold and detoxify Ca^2+^ions. The range of crystal morphologies suggests that *S*. *sclerotiorum* growth and infection controls the form taken by CaOx crystals.

## Introduction

*Sclerotinia sclerotiorum* has been widely studied due to its ability to cause serious disease on many important food crops [1]. The involvement of oxalic acid in pathogenesis by *S*. *sclerotiorum* was noted by de Bary in 1886 [2] and since then many studies have definitively identified it as an important virulence factor for the fungus [3–8]. One of the most convincing demonstrations of its role is the fact that isolates of *S*. *sclerotiorum* which have lost the ability to produce oxalic acid also have lost their ability to infect [4]. In a recent study, *S*. *sclerotiorum* mutants which had completely lost their ability to produce oxalic acid caused limited lesions on *Arabidopsis* leaves, but only after the leaves had been wounded [9].

The importance of oxalic acid to the pathogenesis of *S*. *sclerotiorum* is undoubted, but the mechanisms by which it acts are not well understood. A number of mechanisms have been proposed. Bateman and Beer [10] showed that oxalic acid assisted polygalaturonase enzymes in the *S*. *sclerotiorum* infection process by lowering pH and chelating calcium ions from the cell wall. Guimarães and Stotz [11] found that oxalic acid affected the performance of guard cells in *Vicia faba* leaves, causing them to remain open and leading to wilting of the host. Williams et al. [12] showed evidence that oxalic acid was instrumental in controlling the host cell redox machinery, and hence inducing cell death to the advantage of *S*. *sclerotiorum*. Most recently, Heller and Witt-Geiges [13] postulated that the oxalic acid produced could also be used by *S*. *sclerotiorum* as a means of precipitating calcium ions released as the plant’s cell walls are degraded, protecting it from toxic Ca^2+^ concentrations in plant tissue.

In the course of an investigation into the infection process of *S*. *sclerotiorum* on the stems of *Brassica carinata* (Ethiopian mustard), many calcium oxalate (CaOx) crystals were observed. The crystals clearly varied in shape and size as infection progressed; and CaOx crystals were present on the plant surface, as well as inside stem tissue, and incorporated into hyphal conglomerations within lesions. Although the presence of CaOx crystals after infection by *S*. *sclerotiorum* has previously been shown in cotyledon tissue of *B*. *napus* [14], on the surface of sunflower hypocotyls [13] and around the penetration sites on leaves and roots of carrot [15], the presence of CaOx crystals associated with *S*. *sclerotiorum* infection has not been widely reported, and nearly all detailed studies of the infection process of *S*. *sclerotiorum* do not even mention CaOx crystals [16, 17]. The abundance of CaOx crystals evident in preliminary studies of infection processes made this a puzzling omission. CaOx crystals are produced in most families of higher plants and demonstrate a wide range of morphologies. Knowledge of their formation and function has been studied and reviewed in detail [18, 19]. The four most common morphologies listed by Franceschi and Nakata [18] are prismatic, crystal sand, raphide and druse. According to Franceschi and Nakata [18], most CaOx crystals in plants are formed within cells in special membrane-lined crystal chambers. Factors such as the hydration state of the crystal, the ratio of calcium to oxalate, and the presence of nucleating substances or contaminants, are thought to determine crystal morphology.

Understanding the exact role of oxalic acid in the interaction between *S*. *sclerotiorum* and its crop hosts could allow disease resistance screening for *S*. *sclerotiorum* to be considerably simplified. To date, no *in vitro* assays are available to determine either the aggressiveness of an isolate of *S*. *sclerotiorum* or the degree of resistance in the host. Oxalic acid appears to be an ideal candidate for the development of such an assay. There has been some progress towards demonstrating the link between the amount of oxalic acid produced by individual isolates of *S*. *sclerotiorum* and aggressiveness [5, 6, 20], but results are not consistent. Hosts which are more tolerant to oxalic acid are in general more tolerant to *S*. *sclerotiorum* [21], but in spite of this, attempts to use a plant’s reaction to oxalic acid as a predictor of susceptibility have returned erratic results [22]. Closer examination of the disposition of oxalic acid early in infection, through the observation of CaOx crystals formed on the stem and in the lesion, may reveal factors which could explain the inconsistencies in results of oxalic acid assays. Hence, this study defines the occurrence and nature of CaOx crystals produced during the early interaction between *S*. *sclerotiorum* and *B*. *carinata* and highlights how their occurrence and nature relate to the various stages of the infection process of this pathogen.

## Materials and Methods

The role of CaOx crystals in the interaction between *S*. *sclerotiorum* and *B*. *carinata* was studied in two investigations, one using light microscopy and the other scanning electron microscopy (SEM).

### Investigation 1—Light Microscopy

Two selections of *B*. *carinata* were grown in 16 cm pots in a controlled environment room (CER) maintained at 22°C day/18°C night with a light intensity of 400 μmol m^−2^ s^−1^, 65% relative humidity (RH) with 5 plants per pot. Plants were grown for 6, 8, 10 or 12 weeks before all were simultaneously inoculated. The genotypes used were *B*. *carinata* 054104, ATC 94040 and *B*. *carinata* 054106, ATC 94042. Plants were inoculated with *S*. *sclerotiorum* isolate MBRS-1, which is known to be highly pathogenic [23–26]. Lesion lengths were measured in mm on 30 plants at 72 hpi, and mean lesion lengths were compared by analysis of variance (ANOVA) with GenStat (14^th^ Edition, Lawes Agricultural Trust, Rothamsted Research, UK).

For this study, the inoculation procedure developed by Uloth, You, Cawthray and Barbetti [27], using filter paper discs impregnated with mycelial fragments of *S*. *sclerotiorum*, was used. A cork borer was used to excise five pieces (4 mm diameter) from the actively growing margin of 3-day-old colonies of *S*. *sclerotiorum* grown on potato dextrose agar at 20°C, which were then added to 100 mL of liquid growth medium (PDB 24 g, peptone 10 g, H_2_O 1 L) in 250 mL flasks. The cultures were shaken at 150 rpm at 20°C. After three days, actively growing mycelium was harvested by straining the broth through four layers of cheesecloth and washing with sterile deionized water. The fungal mats obtained were then transferred to 100 mL of the same liquid medium and macerated using a hand held blender for 3 min. The concentration of mycelia in the suspension was measured using a haemocytometer (Superior, Berlin, Germany), and adjusted to 1 x 10^4^ fragments per mL by diluting with the same liquid medium. Clean round discs of filter paper 5mm in diameter (Advantec MFS, Inc. Qualitative No. 2) were added to the broth containing *S*. *sclerotiorum* at least 15 min before inoculation. The filter paper discs were then transferred using forceps to Parafilm strips which were wrapped around the stems of the plants above the first internode, ensuring contact between the impregnated disc and the stem. The moist impregnated paper discs helped maintain high humidity at inoculation sites and were found to reliably initiate infection on the stems. Furthermore, from 24 hours post inoculation (hpi), the discs remained attached to the stem surface during the disease incubation period, making it easy to identify the precise inoculation sites.

After inoculation, plants were returned to the same controlled environmental conditions. Plants were misted with water until leaves were visibly wet and RH in the rooms was increased to 95% night and 90% day, these being the upper limits for humidity possible in these controlled environment rooms. Sections of the stem 1.5 cm long which contained the point of inoculation were harvested at 6, 12, 24, 48 or 72 hpi. Stem sections were decolorized in glass vials containing acetic acid/ethanol/water (2:2:1) for at least 7 d at 22°C until they were observed by light microscopy. At the time of observation, 134 samples were removed from the decolorizing solution and thoroughly rinsed in distilled water. The side of the stem which had been inoculated was stained for 90 s in 0.05% aniline blue, and then rinsed in distilled water. Portions of the stem which stained blue, indicating the presence of mycelium of *S*. *sclerotiorum*, were chosen for examination. A layer containing epidermis and cortical cells were removed using a scalpel, placed on a glass slide, and observed under an Olympus BX51 microscope. Images were recorded using an Olympus DP71 digital photographic system with bright field optics.

**Fig 1:**
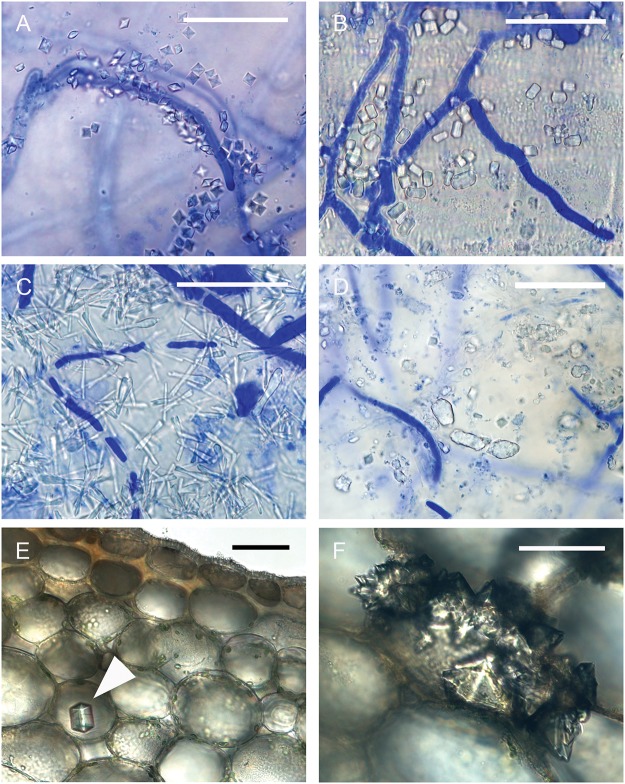
Crystal shapes (72 hpi). Light micrographs illustrating hyphae and crystal shapes observed on *Brassica carinata* infected with *Sclerotinia sclerotiorum* at 72 hours post inoculation: **A-D:** Four crystal shapes on the surface of stems. For these, the epidermis and the first few layers of cortical cells were cut from stems decolorized in acetic acid: ethanol: water (2:2:1), stained in 0.05% aniline blue and observed on glass slides. Photographed using an Olympus BX51 microscope and DP71 digital photographic system. **A:** bipyramidal crystal **B:** short rod crystals **C:** long rod crystals **D:** irregular—crystal sand. **E, F:** The presence of crystals in the stem cortex was revealed when transverse sections 200 μm thick were cut with a Vibratome 3000 sectioning system from stems fixed in glutaraldehyde, then observed under bright field illumination on glass slides. Photographed using Zeiss Axioplan microscope and Zeiss Axiocam digital photographic system. **E:** Tetragonal short rod crystal (arrow) within the stem cortex. **F:** Jagged crystals formed on the surface of a cortical cell. **A, B, C, D, E, F,** Scale bars = 50 μm.

**Fig 2:**
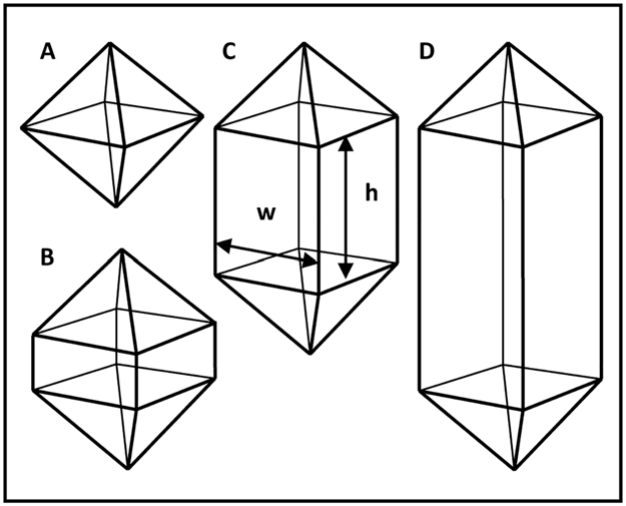
Classification of tetragonal crystals according to their aspect ratio (AR). AR was assessed as the ratio of width to height. **A** and **B:** bipyramidal with AR > 5. **C:** short rod with AR between 5 and 0.5. **D:** long rod, AR < 0.5.

At least four images of each sample were taken from areas where hyphae were visible. The images were examined for the presence of crystals. The proportions of samples in which CaOx crystals were observed on both genotypes of different ages and at different times after inoculation were recorded. A three way analysis of variance was then undertaken using GenStat (14^th^ Edition, Lawes Agricultural Trust, Rothamsted Experimental Station, UK). Crystal shapes were divided into four categories reflecting the descriptions of CaOx crystals found in plants by Franceschi and Nakata [18]. The four shapes were “irregular” (Fig. 1A, corresponding to ‘crystal sand’ but also including other mixed shapes and conglomerates), and three categories of tetragonal crystals, namely “bipyramidal” (Fig. 1B), “short rod” (Fig. 1C) and “long rod” (Fig. 1D). These shapes are illustrated in Fig. 2 and were distinguished on the basis of their aspect ratio, which is the proportional relationship between a crystal’s width (w) and height (h). If the aspect ratio (w:h) was greater than 5:1, the crystals were considered “bipyramidal”. If the aspect ratio was between 5:1 and 1:2, they were designated “short rod”, and if the ratio was less than 1:2 they were classified as “long rod”. The “long rod” category included needle shaped raphides, even though the tetragonal crystal structure for these needles was not clear. The percentage of each crystal shape present was recorded for each sample, and a one way analysis of variance undertaken using GenStat.

### Investigation 2—Scanning Electron Microscopy

Two selections of *B*. *carinata* were grown in 14 cm pots in a growth room maintained at 22°C day/18°C night with a 12 hour photoperiod and a light intensity of 400 μE m^−2^ s^−1^, and at 65% RH for 5 weeks, with 4 plants per pot. Selections were a susceptible selection, *B*. *carinata* SMP3-82 and a resistant selection, *B*. *carinata* 054113 [23], that are known to be consistently different in their susceptibility to *S*. *sclerotiorum* [27]. Plants were inoculated as described above, with filter paper discs soaked in *S*. *sclerotiorum* inoculum. At the time of inoculation, plants were approximately 30 cm tall with a mean stem diameter of 7 to 8 mm. Two isolates were used, viz. MBRS-1 (generally highly virulent) and WW3 (generally weakly virulent) on *B*. *carinata* stems [23]. Two plants in each treatment x replication were mock inoculated with filter paper discs which had been soaked in growth media with no added fungus while two others were mechanically injured by piercing with the tip of fine forceps before being wrapped in Parafilm; in order to demonstrate that symptoms resulted solely from *S*. *sclerotiorum* and not from any physical damage associated with inoculation.

After inoculation, plants were placed in controlled environmental conditions at either 25/21°C or 18/14°C. They were misted with a very fine water spray until leaves were visibly wet, and relative humidity in the rooms was maintained at 95% night and 90% day. After 24, 48 and 72 h, portions of stem 1.5 cm long were cut from the plants and immediately immersed in 2.5% glutaraldehyde in phosphate buffered saline (PBS). Samples were held at 4°C until processed. Small portions containing infection sites were cut from the surface of the stems to a depth of approximately 3 mm. These were dehydrated in a graded series of ethanol (30–100% anhydrous) solutions using a PELCO Biowave microwave fitted with PELCO coldspot, at 250 Watts for 40 s. Samples were then critical point dried in a Polaron E3000 critical point drier, mounted on aluminium pin mounts with carbon tabs, and coated with 3 nm platinum and 10 nm carbon.

SEM imaging and qualitative energy dispersive X-ray (EDS) analyses were conducted in a field emission SEM (Zeiss Supra) fitted with an 80 mm Silicon Drift Detector EDS detector. All imaging was done at 5 kV. Spectra were acquired in spot mode at 15kV from crystals of interest using AZtec software (Oxford Instruments). Spectra peaks for C and Pt in these spectra reflect the sample coating.

Examples of each of the different crystal shapes and conglomerates described in this paper were qualitatively analyzed using EDS microanalysis to ascertain their elemental composition. Additional crystal shapes were revealed in the SEM imaging, and these were classified according to the schema of Franceschi and Nakata [18].

Transverse sections 200 μm thick were also cut from stem sections that had been fixed, using a Vibratome 3000 sectioning system fitted with razor blades. These sections were either processed for SEM as above or mounted in water on glass slides and observed without staining under bright field illumination (Zeiss Axioplan microscope) and photographed using a digital camera (Zeiss Axiocam).

## Results

### Stem Surface—Light Microscopy

**Fig 3:**
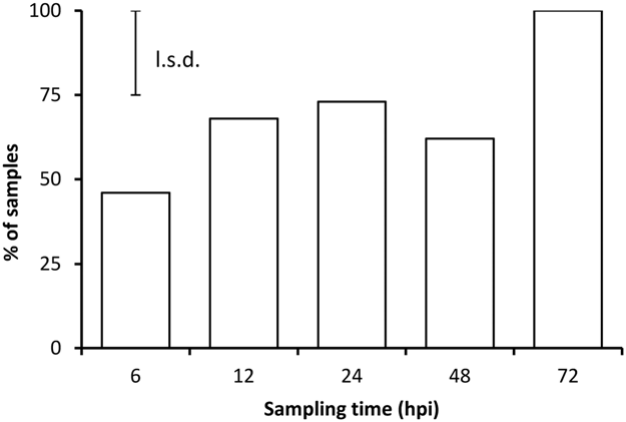
Occurrence of calcium oxalate crystals. Percentage of samples containing calcium oxalate crystals at 6, 12, 24, 48 and 72 hours post inoculation (*n* = 134. *P* = 0.002, l.s.d. = 25%).

*S*. *sclerotiorum* successfully infected stems of *B*. *carinata* inoculated with filter paper discs impregnated with hyphal fragments of *S*. *sclerotiorum*. A few hyphae were visible on the surface of the stems close to the edge of the filter paper discs at 6 hpi in plants of all ages. The amount of hyphae on the stem surface subsequently increased, with the first appressoria visible at 12 hpi. Infection cushions were observed by 24 hpi and bundles of parallel hyphae could be seen extending from underneath infection cushions under the plant epidermis by 48 hpi. By 72 hpi, dark sunken lesions containing hyphal masses were evident on the stems, and a few hyphae were present on the stem outside of the lesion borders. Crystals were associated with hyphae of *S*. *sclerotiorum* and on the surface of inoculated *B*. *carinata* from 6 hpi onwards (Fig. 3). No hyphae or crystals were observed on controls inoculated only with growth medium or following mechanical injury of stems. Crystals were observed on 46% of all samples at 6 hpi, increasing to 100% of samples by 72 hpi (Fig. 3) with an overall mean incidence of 72% (l.s.d.(0.05) = 25%, *P* = 0.002). The proportion of samples where crystals were observed was not affected by the age of the plant when inoculated or by the host genotype (*P* > 0.05).

**Fig 4:**
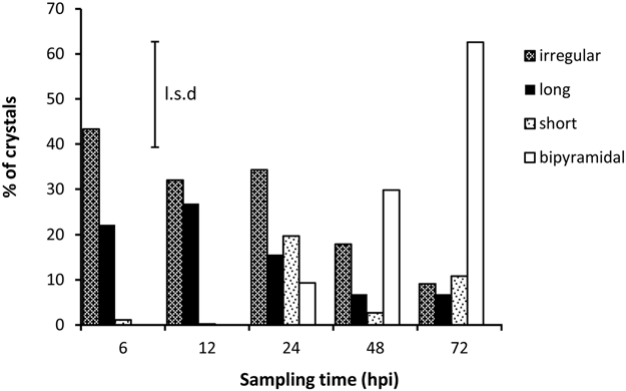
Changes in crystal shape over time. Percentage of calcium oxalate crystal-containing samples in which each of four differently shaped crystal forms were observed at 6, 12, 24, 48 and 72 hours post inoculation (*n* = 96, *P* = 0.001, l.s.d. = 23.4%).

The mix of different crystal shapes varied with the time after inoculation (Fig. 4, crystal type x hours after inoculation interaction *P* <0.001, l.s.d. = 23.4%). Crystals of irregular shape dominated at 6, 12 and 24 hpi (found in 43%, 32% and 34% of samples containing crystals). Long rods were also common early in pathogenesis (22% of samples at 6 hpi), but by 72 hpi were only present in 7% of samples. Short rods of intermediate length appeared 24 hpi (20% of samples), and remained present in small numbers (3–11% of samples) until 72 hpi. No bipyramidal crystals were observed until 24 hpi, but by 72 hpi, 63% of samples contained bipyramidal crystals. The size of crystals also varied widely, and ranged from approximately 0.5 μm to 25 μm in width. Bipyramidal crystals were typically between 2 μm to 5 μm in width, but examples as small as 0.7 μm were observed.

There were no significant differences in the level of disease between *B*. *carinata* 054104 and 054106 at 72 hpi under these particular controlled environment conditions (*P* = 0.947), and both had small lesion length (mean for both genotypes 1.3 mm).

### Stem Surface—Scanning Electron Microscopy

**Fig 5:**
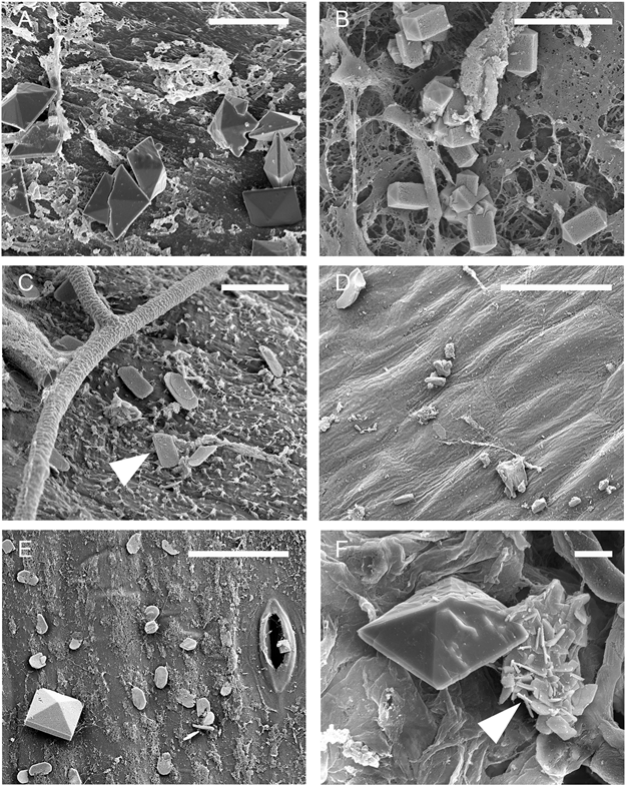
Crystal shapes and arrangements. Scanning electron micrographs illustrating hyphae and crystal shapes and arrangements observed on the surface of stems of *Brassica carinata* 72 hours post inoculation with *Sclerotinia sclerotiorum* **A:** Bipyramidal. **B:** Short rod. **C:** Oval plates. **D:** Crystal sand. **E:** Mixture of bipyramidal and oval plates. **F:** Bipyramidal and druse crystals (arrow). **A, B, C, F,** Scale bars = 5 μm. **D, E,** Scale bars = 50 μm.

**Fig 6:**
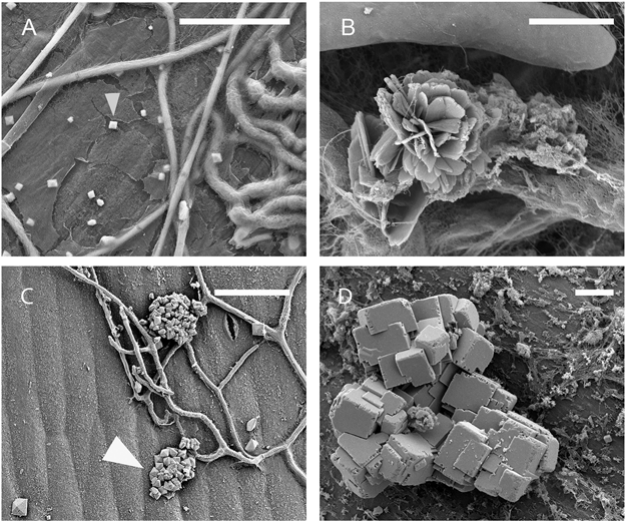
Crystal locations and arrangements. Scanning electron micrographs illustrating crystal locations and arrangements observed on stems of *Brassica carinata* infected with *Sclerotinia sclerotiorum*. Unless otherwise noted, images were taken 72 hours post inoculation. **A:** Bipyramidal crystals on stem surface loosely associated with *S*. *sclerotiorum* hyphae at 24 hours post inoculation. **B:** Flower-like druse. **C:** Bipyramidal crystal cluster (arrow) at 48 hours post inoculation. **D:** Complex of rectangular crystals. **B, D**, Scale bars = 5 μm **A, C**, Scale bars = 50 μm.

**Fig 7:**
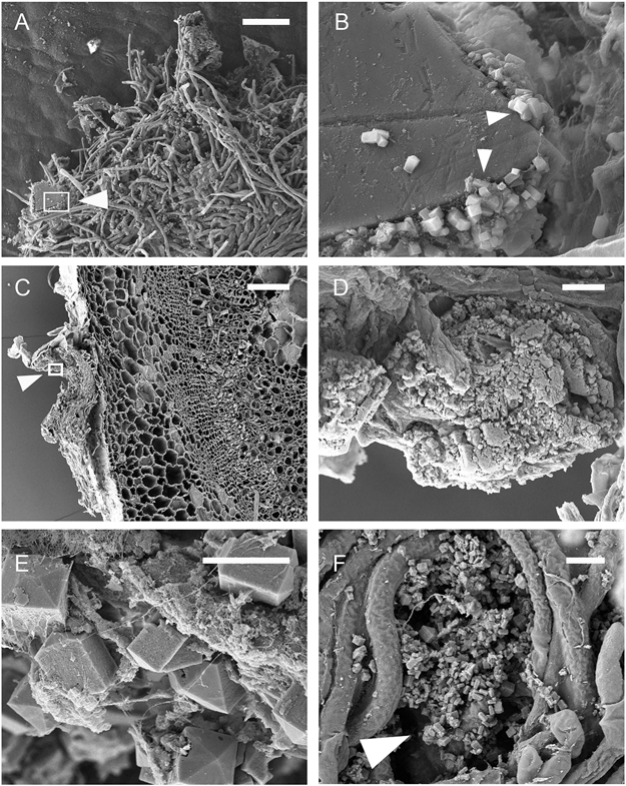
Complex crystal structures. Scanning electron micrographs illustrating crystal locations, arrangements, hyphal masses and crystal conglomerates as observed on stems of *Brassica carinata* infected with *Sclerotinia sclerotiorum*. Unless otherwise noted, images were taken 72 hours post inoculation. **A:** Large crystal incorporated into an infection cushion at 48 hours post inoculation showing location of the detail shown in B (arrow). **B:** Small tetragonal crystals formed along the edges of a larger crystal (arrows). **C:** 200 μm thick transverse section showing the hyphal mass and the location of the detail illustrated in D (arrow). **D:** CaOx conglomerate. **E:** Conglomerate containing short rod crystals. **F:** Abundant short/long rod and bipyramidal crystals forming within the hyphal mass. **B, D, E, F,** Scale bars = 5 μm. **A, C,** Scale bars = 50 μm.

SEM revealed additional details in relation to the occurrence, distribution, chemical composition and shape of CaOx crystals formed in association with *S*. *sclerotiorum* infection of stems of *B*. *carinata* (Fig. 5, 6 and 7). SEM confirmed the ubiquitous presence of CaOx crystals on stems of *B*. *carinata* infected by *S*. *sclerotiorum*. Examples ranged from tiny crystals (< 0.5 μm) of irregular shape embedded in mucilage (crystal sand) up to large (up to 40 μm) highly organized arrangements. Crystals were observed in every infected sample (*n* = 22) observed under the SEM. The presence of regular tetragonal shaped crystals ranging in width from approximately 2 to 10 μm was common. All the shapes that had been observed by light microscopy were seen with the SEM with the exception of raphides (Fig. 5A, B and D). In addition, flat plates were often observed (Fig. 5C and E). These were predominantly oval. It was not unusual to see a variety of crystal shapes in one sample (Fig. 6E and F) but in general more than 90% of crystals belonged to the dominant shape.

**Fig 8:**
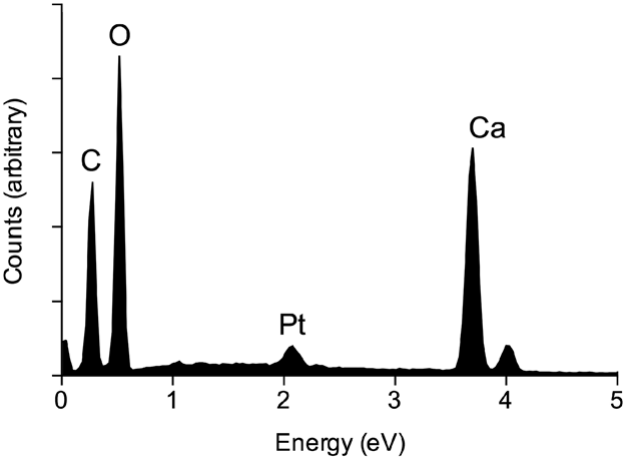
Composition of crystals. Qualitative energy dispersive X-ray (EDS) spectrum showing typical elemental composition of crystals. Samples were coated with carbon and platinum.

At 24 hpi, crystals were mainly on the plant surface and only loosely associated with hyphae (Fig. 6A), but by 48 hpi the crystals were incorporated within hyphal masses (Fig. 7A), and by 72 hpi large conglomerations of crystals in a matrix were present within the hyphal mass (Fig. 7C, E and F). Many examples of complex crystal formations similar to druses (roughly spherical objects with many crystal facets radiating from a central core [18]) were observed (Fig. 5F and 6B), with the majority being ‘flower-like’ arrangements of flat crystals (Fig. 6B). Other complex arrangements of crystals were also observed (Fig. 6C and D). By 72 hpi, bipyramidal crystals and conglomerates were the predominant forms of CaOx present. All crystal types produced dominant EDS spectral peaks for calcium and oxygen. The spectrum in Fig. 8 is representative for crystals analyzed, with little variation between individual spectra. This is consistent with a composition of CaOx [28, 29].

Measurement of lesion size demonstrated that *B*. *carinata* SMP3-82 was consistently more susceptible to infection by *S*. *sclerotiorum* than *B*. *carinata* 054113 (mean lesion lengths at 72 hpi were 6.1 mm and 2.9 mm, respectively). However, no clear difference in the distribution or occurrence of CaOx crystals was observed between the two *B*. *carinata* varieties. Long rods were observed only in samples collected at 12 or 24 hpi, and none were seen in samples collected later. The dominant crystal shapes observed in each sample were recorded. Bipyramidal crystals dominated in 8 samples, short rods in 6 samples, long rods in 3 samples, oval plates in 3 samples and 2 samples had no dominant form, with a mixture of shapes.

### Stem Cortex—Vibratome

A variety of structures consistent with CaOx crystals were observed within the cortex of a small number of stems (<10%) when vibratome sections from plants 72 hpi were observed under bright field illumination. In several examples, crystals appeared as jagged protuberances on the surface of cortical cells (Fig. 1E) but individual tetragonal crystals were also observed (Fig. 1F).

## Discussion

This study has clearly shown that CaOx crystals are present on the surface of *B*. *carinata* stems infected by *S*. *sclerotiorum* as early as 6 hpi. This is well before the development of any infection structures by the fungus and highlights that the secretion of oxalic acid commences before hyphal penetration. This contrasts with the findings of Heller and Witt-Geiges [13], who did not observe CaOx crystals on sunflower at 12 or 24 hpi but only after 36 hpi. In fact, they were only able to demonstrate the presence of CaOx crystals after nearby hyphae and infection cushions were dead and without function, whereas, in contrast, we found many instances where CaOx crystals were clearly associated with actively growing hyphae. Garg et al. [14] only found CaOx crystals in the tissue of *B*. *napus* 6 days post-inoculation; and on sunflower cotyledons, an increase in oxalic acid production by *S*. *sclerotiorum* was only observed once 40% of the leaf was destroyed [30]. In our study, numerous crystals were present on the surface of the stem early in the infection process (e.g., by 12 hpi) and not always immediately adjacent to the fungal hyphae. This suggests that oxalic acid produced by the fungus dispersed away from the hyphae on the surface of the stem. The failure to demonstrate early production of oxalic acid in some studies may be due to the combination of isolate and temperature used. Uloth, You, Cawthray and Barbetti [27] demonstrated differing levels of oxalic acid production across several isolates of *S*. *sclerotiorum* and also when temperature was varied. If the level of production of oxalic acid was lower overall, it would increase the difficulty of detecting oxalic acid produced early in the infection process. Once *S*. *sclerotiorum* had entered the plant, crystals were also clearly visible in stem cross sections at 72 hpi, both as regularly shaped tetragonal crystals outside cortical cells and at other times formed directly on the surface of cells in jagged formations. Both of these manifestations of CaOx are consistent with the crystals revealed within cotyledon tissue when Garg et al. [14] imaged stained GMA sections of *B*. *napus* infected with *S*. *sclerotiorum*. The formation of CaOx crystals on the surface of individual cortical cells provides support for the theory that part of the function of oxalic acid is to chelate calcium ions from the cell wall [10], due to the close association with the cell wall and the lack of crystals inside the cells. We demonstrated that CaOx was only produced on the stems of *B*. *carinata* in the presence of *S*. *sclerotiorum*, through the use of control comparison plants with or without physical damage. The presence of CaOx crystals on the stem of the host from very early in the infection process suggests the possibility of using the presence of tetragonal crystals to diagnose *S*. *sclerotiorum* infection at its earliest stages.

The morphology of crystals found as a result of the *S*. *sclerotiorum*/*B*. *carinata* interaction was diverse. Tetragonal crystals were prominent, with the prismatic edges varying greatly in size (0.7–10 μm). There were also flat plate, raphide, crystal sand, and druse morphologies, as well as large conglomerates of CaOx crystals observed. In mature hyphal masses, bipyramidal crystals and conglomerates were the predominant forms of CaOx found. This is the first time CaOx crystals have been reported on *B*. *carinata* infected with *S*. *sclerotiorum* and our results confirm and extend the limited previous work with *S*. *sclerotiorum* on other hosts and also for other fungal pathogens. For example, previous studies with *S*. *sclerotiorum* have demonstrated the presence of bipyramidal and plate shaped CaOx crystals on *B*. *napus* [13] and short rod tetragonal CaOx crystals on carrots [15]. The fungi *Rhizoctonia*, *Gilbertella persicaria* and several other basidiomycetes have been associated with the formation of CaOx crystals of varying shapes including bipyramidal, plate shape, sphere, raphide and long rod morphologies [28, 29, 31]. X-ray energy-dispersive emission spectra showed that the major elements present in all the crystal shapes we observed were calcium and oxygen, confirming that these crystals were indeed CaOx.

It is important to note that the formation of CaOx crystals by *S*. *sclerotiorum* differs from its production by plants in several important respects. Firstly, we found all of the main shapes listed by Franceschi and Nakata [18] in one pathosystem and this contrasts with their summary of CaOx production in plants, where each plant species is usually associated with a single main crystal morphology. Secondly, in our study the CaOx crystals are formed outside the hyphae of *S*. *sclerotiorum* on the stem surface and on the outside of both hyphae and also the outside of plant cells when formed within the stem cortex. The frequency with which the tetragonal prism morphology was observed, however, does imply that *S*. *sclerotiorum* has some control of the morphology of the CaOx crystals. How this control is achieved by *S*. *sclerotiorum* and other fungi is an area worthy of future study. In other organisms, it is recognized that the process of crystal formation is not a simple physical-chemical process and that control over crystal shape may be achieved through the influence of biomacromolecules acting as nucleators, matrixes or moulds [32]. It is likely in the case of *S*. *sclerotiorum*/*B*. *carinata* that the unique chemical environment produced by the interaction of the fungus and host is driving the formation of particular crystal shapes. Crystal-containing microbodies have been observed in the hyphal tips of *S*. *sclerotiorum* [6], and CaOx is known to precipitate in vacuoles within the fungus [13]. These may act as seed crystals which subsequently control the external formation of CaOx crystals.

The aspect ratio of the tetragonal crystals in this study varied greatly, and this changed with the stage of infection. This is a likely indicator of the varying amount of oxalic acid produced by the fungus and the concentration of Ca^2+^ ions free on the plant surface as infection progresses. This conclusion is supported by a study of factors that can change the crystallisation of CaOx [32], where an increase in the levels of oxalic acid or a decrease in the amount of Ca^2+^ compared to oxalic acid decreased the length of CaOx crystals. Both of these factors may be involved in the changes we observed in the dominant crystal shape from raphides or long rods to bipyramidal as infection by *S*. *sclerotiorum* progressed. The change in dominant shape suggests that the amount of CaOx on the stem surface associated with hyphae of *S*. *sclerotiorum* increases with time after infection, and this may also be an indicator of abundant Ca^2+^ liberated from cell walls in the early stages of infection. Billon-Grand, Rascle, Droux, Rollins and Poussereau [30] also reported an increase in the concentration of oxalic acid in the plant as infection progressed. Smaller amounts of CaOx on the stem initially could be due to the early sequestration of oxalate by the plant at the beginning of infection as argued by Heller and Witt-Geiges [13]; or due to low levels of production by *S*. *sclerotiorum* early in the infection process. Further research is needed to clarify the relative importance of these possible mechanisms. Our study highlights the dynamism of the occurrence of oxalate over time as infection progresses.

Evidence from the current and previous studies [10–13] is mounting that oxalic acid plays multiple critical roles in the pathogenesis of *S*. *sclerotiorum*. The findings of Liang, Liberti, Li, Kim, Hutchens, Wilson and Rollins [9] that the progress of disease caused by *S*. *sclerotiorum* is greatly reduced in the absence of oxalic acid (even after wounding) also demonstrate its importance, while simultaneously showing that oxalic acid is not the only determinant of pathogenicity.

To the best of our knowledge, the formation of large conglomerates of CaOx crystals cemented together in mature lesions caused by *S*. *sclerotiorum* has not previously been reported. These conglomerates may be a strategy of the fungus to retain and detoxify Ca^2+^ ions in ‘waste dumps’ of CaOx, in order to maintain favourable conditions at the growing margin of the fungus [17, 31].

While the current study showed that the occurrence and shape of CaOx crystals varied depending on time after inoculation, no effect of the genotype of *B*. *carinata* on the occurrence or predominance of one or more particular crystal morphologies was demonstrated. This contrasts with the work of Garg, Li, Sivasithamparam, Kuo and Barbetti [14], who detected more CaOx crystals in cross sections of susceptible infected *B*. *napus* cotyledons than in resistant cotyledons. This appeared to be due to greater growth of the fungus within the susceptible cotyledons. Our inability to demonstrate differences is disappointing given that the original aims of the study were to associate the pattern of CaOx crystal occurrence with resistance in *Brassica* stems. The future development of improved methods which would allow crystals to be visualised in 3D and quantified *in situ* may allow differences to be detected. It is noteworthy that most studies on *S*. *sclerotiorum* do not mention the presence of CaOx crystals. The reasons for this are still unclear, especially considering the ubiquitous presence of the crystals in our study. Specific investigation with a wide range of hosts would help to elucidate whether CaOx crystals only form abundantly when *S*. *sclerotiorum* attacks particular hosts. For example, plants such as *B*. *carinata* and carrots (the other hosts on which large numbers of crystals have been reported in association with *S*. *sclerotiorum* [15]) may induce more crystals than plants such as *B*. *napus*. It is also possible that the particular combination of inoculation, decolourization and staining used in the present study, different to previous reported studies, was particularly effective for visualising CaOx crystals.

## Conclusions

CaOx crystals appeared on the stems of *B*. *carinata* infected with *S*. *sclerotiorum* as early as 6 hpi, well before the development of any infection structures by the fungus, highlighting that the secretion of oxalic acid commences before hyphal penetration. The formation of CaOx crystals on the surface of individual cortical cells, closely in contact with the cell wall, supports the theory that part of the function of oxalic acid is to chelate calcium ions from the cell wall. That crystals are present on the surface of infected *B*. *carinata*, within the stem cortex and are closely associated with the developing fungal hyphae, together strongly support the concept that oxalic acid is active throughout and critical to the infection process of *S*. *sclerotiorum*. Our results show that the amount of oxalic acid present on the stem surface increases dramatically as infection progresses and that *S*. *sclerotiorum* exerts significant control over the form taken by CaOx crystals. This study provides evidence of these critical chemical processes taking place during the infection of *B*. *carinata* by *S*. *sclerotiorum*. The diversity of crystals and complex crystal structures formed in this pathosystem are indicators of the complexity of the chemical environment present on and within stems in the presence of *S*. *sclerotiorum*.
